# A longitudinal study highlights shared aspects of the transcriptomic response to cardiogenic and septic shock

**DOI:** 10.1186/s13054-019-2670-8

**Published:** 2019-12-19

**Authors:** Daniele Braga, Matteo Barcella, Antoine Herpain, Federico Aletti, Erik B. Kistler, Bernardo Bollen Pinto, Karim Bendjelid, Cristina Barlassina

**Affiliations:** 1 Dipartimento di Scienze della Salute, Università degli Studi di Milano, 20142 Milano, Italy; 2 Fondazione Filarete, 20139 Milano, Italy; 3 Department of Intensive Care, Hôpital Erasme, Université Libre de Bruxelles, Brussels, Belgium; 4 Department of Bioengineering, University of California San Diego, La Jolla, CA USA; 5 Department of Anestesiology & Critical Care, University of California, San Diego, USA; 6 Department of Anaesthesia, Pharmacology and Intensive Care, Geneva University Hospitals, Geneva, Switzerland

**Keywords:** Septic shock, Cardiogenic shock, Critical illness, Circulatory shock, RNA-Seq, PRR, Immunoglobulin

## Abstract

**Background:**

Septic shock (SS) and cardiogenic shock (CS) are two types of circulatory shock with a different etiology. Several studies have described the molecular alterations in SS patients, whereas the molecular factors involved in CS have been poorly investigated. We aimed to assess in the whole blood of CS and SS patients, using septic patients without shock (SC) as controls, transcriptomic modifications that occur over 1 week after ICU admission and are common to the two types of shock.

**Methods:**

We performed whole blood RNA sequencing in 21 SS, 11 CS, and 5 SC. In shock patients, blood samples were collected within 16 h from ICU admission (T1), 48 h after ICU admission (T2), and at day 7 or before discharge (T3). In controls, blood samples were available at T1 and T2. Gene expression changes over time have been studied in CS, SS, and SC separately with a paired analysis. Genes with *p* value < 0.01 (Benjamini-Hochberg multiple test correction) were defined differentially expressed (DEGs). We used gene set enrichment analysis (GSEA) to identify the biological processes and transcriptional regulators significantly enriched in both types of shock.

**Results:**

In both CS and SS patients, GO terms of inflammatory response and pattern recognition receptors (PRRs) were downregulated following ICU admission, whereas gene sets of DNA replication were upregulated. At the gene level, we observed that alarmins, interleukin receptors, PRRs, inflammasome, and DNA replication genes significantly changed their expression in CS and SS, but not in SC. Analysis of transcription factor targets showed in both CS and SS patients, an enrichment of CCAAT-enhancer-binding protein beta (CEBPB) targets in genes downregulated over time and an enrichment of E2F targets in genes with an increasing expression trend.

**Conclusions:**

This pilot study supports, within the limits of a small sample size, the role of alarmins, PRRs, DNA replication, and immunoglobulins in the pathophysiology of circulatory shock, either in the presence of infection or not. We hypothesize that these genes could be potential targets of therapeutic interventions in CS and SS.

**Trial registration:**

ClinicalTrials.gov, NCT02141607. Registered 19 May 2014.

## Background

Circulatory shock is a common life-threatening condition in critical care that affects approximately one third of all patients admitted to the intensive care unit (ICU), with accompanying high mortality [1, 2]. Circulatory shock is characterized by systemic arterial hypotension associated with tissue hypoperfusion, acidemia, and increased blood lactate levels that reflect resultant tissue hypoxia, which in turn may lead to multi-system organ failure and eventual death [3]. Septic shock (SS) and cardiogenic shock (CS) are two forms of circulatory shock with different etiologies but similar end-organ effects. SS, the most common form of shock in the ICU, is a cardiovascular complication of sepsis resulting from a complex interplay of overwhelming systemic inflammation and paradoxical lack of host response, resulting in recalcitrant vasoplegia and variably some degree of cardiac dysfunction [4]. CS, on the other hand, results from acutely depressed cardiac output secondary to cardiac pump failure, with myocardial infarction as its most common cause and systemic inflammation evolving later in response. Both types of circulatory shock are associated with high mortality: 30% for SS [5] and 40% in CS [6]. The molecular mechanisms of SS have been widely studied with –omic approaches in patient cohorts [7–14]. Conversely, the molecular factors involved in CS have been poorly studied and up to now no –omic data are available. We performed a longitudinal study with a time course RNA sequencing analysis in order to explore the transcriptome in the whole blood of septic and cardiogenic shock patients during the first 7 days of ICU stay. The purpose of this pilot analysis was to highlight the transcriptomic signatures common to CS and SS, using septic patients without shock as controls.

## Methods

### Study design and participants

This study was part of the multicenter prospective observational trial ShockOmics (ClinicalTrials.gov identifier: NCT02141607, EU grant #602706). Patients were recruited from the ICUs of the Hôpitaux Universitaires de Genève, Université de Genève (Geneva, Switzerland), and Hôpital Erasme, Université Libre de Bruxelles (Brussels, Belgium). The clinical protocol was approved by the ethical committees of the two participating institutions, and informed consent was obtained from the patients or their representatives. In the present study, we included consecutive adult (> 18 years old) patients admitted to the ICU for SS or CS with a SOFA score at admission > 6 and arterial lactate > 2 mmol/L, as previously described [15]. Moreover, patients had to have blood samples, for the analysis of gene expression, collected at three time points: T1, within 16 h of ICU admission; T2, 48 h after study enrollment; and T3, on day 7 from ICU admission or before discharge from the ICU. Exclusion criteria were expected death within 24 h of ICU admission, transfusion of ≥ 4 units of packed red blood cells or infusion of ≥ 1 unit of fresh frozen plasma, active hematological malignancy, metastatic cancer, chronic immunosuppression, pre-existing end-stage renal disease requiring renal replacement therapy, recent cardiac surgery, Child-Pugh C cirrhosis, and terminal illness.

Septic patients without shock were included in the study as negative controls. Admission criteria for these patients were a proven or clinically suspected infection, associated with at least one organ dysfunction but the cardiovascular system, as indicated by SOFA score, and lactate levels < 2 mmol/L. In these patients, blood samples were collected at two time points: T1, within 16 h of ICU admission, and T2, 48 h after study enrollment.

### Analysis of laboratory and clinical variables

Laboratory and clinical variables measured at the available time points were analyzed using a linear mixed model accounting for fixed effects of time, gender, age, and random effects of patients. We identified the differences between CS, SS, and SC with ANOVA. Demographic variables and variables with one measurement at a single time point were compared using the Wilcoxon rank-sum test or Fisher exact test for categorical variables.

### Blood collection and RNA extraction

Peripheral blood was collected at the time points foreseen by the study design in EDTA tubes with 400 μL of 2× Denaturing solution (Ambion, Austin, TX, USA) and stored at − 20 °C. Total RNA was extracted from 800 μL of blood with the MirVana Paris Kit and treated with Turbo DNA-free Kit (Ambion). RNA concentration was estimated with a Nanoquant Infinite M200 instrument (Tecan, Austria). RNA quality was assessed on an Agilent Bioanalyzer using the RNA 6000 Nano Kit (Agilent, Santa Clara, CA, USA), and samples with RNA integrity number > 7.5 were considered acceptable for processing.

### Library preparation

We prepared sequencing libraries with the TruSeq Stranded Total RNA with Ribo-Zero Globin Kit (Illumina, San Diego, CA, USA) using 800 ng of total RNA input. Final libraries were validated with the Agilent DNA1000 kit and sequenced on a HiSeq2500 platform, producing 50 × 2 base paired-end reads.

### Sequencing data analysis

We aligned high-quality paired-end reads to the human reference genome (GRCh38) using STAR (version 2.5.2b) [16], and we selected only uniquely mapping reads. We assigned sequencing reads to genes with featureCounts (version 1.5.1) [17] using the gencode (version 25) primary assembly gene transfer file (GTF) as a reference annotation file for genomic feature boundaries.

### Exploratory and differential expression analysis

DESeq2 [18] package built-in functions were used for data preprocessing, exploratory data analysis and analysis of differential gene expression. We studied gene expression changes over time in CS and SS patients and SC separately with a paired analysis, comparing T1 to T2 in SS and SC and T1 to T3 in CS and SS. Genes with padj < 0.01—Benjamini-Hochberg multiple test correction (FDR)—were considered differentially expressed (DEGs) and used for downstream analysis.

### Gene set enrichment analysis

We first performed gene set enrichment analysis (GSEA) [19] to identify the biological processes enriched in CS, SS, and SC. For this purpose, the lists of genes were ranked for log_2_FC (T2 vs T1 for SS and SC, and T3 vs T1 for CS) and used as input for GSEA, together with the gene set database c5.bp.v6.2.symbols.gmt. As the first step, we selected significant GSEA terms (FDR < 0.1) in at least one type of shock and filtered for the number of DEGs ≥ 5 in CS and SS. GOs common to the two types of shock, as well as specific of shock type (CS vs SS), were manually selected from the dataset. Starting from the identified GO terms, we selected a list of genes showing significant modulation over time in CS and SS, but not modulated in SC.

### Analysis of transcriptional regulators

Gene set enrichment analysis of transcription factor targets (TFT) was performed on the lists of CS and SS genes ranked for log_2_FC expression between T1 and T3. Enriched gene sets were filtered for FDR < 0.1 and number of DEGs ≥ 10.

## Results

### Patients

Seventy-nine shock patients were recruited between November 2014 and March 2016 in the frame of ShockOmics trial. Twenty-one septic shock and 11 cardiogenic shock patients (Additional file 1) were eligible for the present study, after excluding 16 patients who did not meet the inclusion criteria and 31 patients who did not have blood samples collected at the three time points (Additional file 2). Five septic patients, not developing shock, were recruited as controls (SC) and followed for two time points.

At ICU admission, there were no significant demographic differences between the CS and SS group, including age, BMI, severity of illness (APACHEII), and needs for norepinephrine. In both groups, about 20% of patients died later in the period from the second to the fourth week, after study enrollment. SOFA and lactate levels decreased over the measured 1 week of ICU stay in all patients. We assessed SOFA score in patients classified according to mortality (alive or dead at 28 days), showing that a decreasing trend of SOFA can be appreciated also in patients who died (Additional file 3). Laboratory results from the blood collected at the three time points of interest, however, revealed significant differences between cardiogenic and septic shock patients for C-reactive protein (CRP) level, lymphocyte count, hematocrit, and fibrinogen (*p* < 0.05) (Table 1). Diastolic blood pressure, mean arterial pressure, and temperature were significantly different as well (Table 1). SC compared to SS patients showed significant lower severity scores (APACHE II and SOFA) at ICU admission (Table 2) and did not develop any circulatory failure later during the course of the ICU stay. Clinical and laboratory variables in SC and SS patients are available in Additional file 7.

**Table 1:** Clinical and laboratory variables with follow-up for 1 week

Clinical variable	Shock	T1	T2	T3	*p* value
Heart rate, bpm	CS	87 (9)	90 (15)	92 (23)	0.69532
	SS	91 (22)	81 (13)	88 (22)	
Systolic blood pressure, mmHg	CS	84 (11)	105 (24)	113 (34)	0.31639
	SS	83 (11)	97 (16)	110 (17)	
Diastolic blood pressure, mmHg	CS	51 (7)	52 (9)	63 (17)	0.01147
	SS	45 (4)	49 (5)	53 (12)	
Mean arterial pressure, mmHg	CS	61 (6)	69 (14)	79 (21)	0.04505
	SS	57 (5)	64 (7)	72 (12)	
Respiratory rate, rpm	CS	21 (5)	26 (7)	27 (3)	0.82505
	SS	25 (8)	25 (6)	27 (8)	
PaO_2_, mmHg	CS	92 (44)	79 (15)	77 (12)	0.17879
	SS	85 (17)	76 (18)	70 (11)	
PaCO_2_, mmHg	CS	35.0 (13.7)	39.3 (6.5)	38.8 (5.6)	0.10633
	SS	44.1 (13.0)	40.3 (8.5)	38.5 (6.9)	
SvcO_2_%	CS	64 (11)	67 (5)	86 (7)	0.49098
	SS	73 (6)	69 (8)	61 (17)	
FiO_2_	CS	0.59 (0.30)	0.35 (0.08)	0.33 (0.08)	0.87275
	SS	0.52 (0.20)	0.34 (0.10)	0.33 (0.08)	
PaO_2_/FiO_2_	CS	189 (109)	239 (70)	250 (73)	0.51411
	SS	194 (103)	245 (89)	229 (64)	
Temperature, °C	CS	36.7 (1.5)	37.3 (0.8)	36.7 (1.0)	0.01178
	SS	37.6 (0.9)	37.5 (0.9)	37.5 (1.3)	
Urine output, mL/day	CS	1492 (851)	2184 (926)	1963 (1323)	0.18808
	SS	1494 (831)	2272 (1057)	2591 (1219)	
Fluid balance, mL	CS	1427 (1417)	197 (1104)	− 17 (1267)	0.59677
	SS	2591 (1958)	411 (1529)	− 815 (1358)	
HCO_3_, mmol/L	CS	18.1 (3.9)	26.0 (3.9)	27.6 (3.6)	0.57272
	SS	19.8 (3.9)	25.1 (4.8)	27.0 (5.0)	
Norepinephrine, μg/(kg min)	CS	0.23 (0.2)	0.20 (0.22)	0.16 (0.13)	0.58250
	SS	0.35 (0.29)	0.23 (0.34)	0.05 (0.02)	
Bilirubin, mg/dL	CS	1.1 (0.6)	0.9 (0.4)	0.8 (0.6)	0.56664
	SS	1.6 (1.8)	1.2 (1.4)	1.0 (1.2)	
Glycemia, mg/dL	CS	218.2 (73.6)	146.9 (25.1)	140.9 (54.4)	0.37518
	SS	183.9 (84.8)	142.8 (37.9)	129.8 (34.3)	
Prothrombin time INR	CS	1.5 (0.8)	1.2 (0.5)	1.1 (0.1)	0.09433
	SS	1.3 (0.3)	1.1 (0.2)	1.1 (0.2)	
Fibrinogen, g/L	CS	4.11 (1.46)	4.34 (2.33)	7.15 (1.68)	0.02844
	SS	5.32 (2.14)	6.35 (2.13)	6.81 (1.53)	
CRP value, mg/L	CS	62.8 (49.3)	149.7 (74.8)	75.5 (39.2)	0.00001
	SS	248.5 (140.6)	248.3 (112.5)	120.4 (71.0)	
Creatinine, mg/dL	CS	1.4 (0.5)	1.3 (0.6)	1.0 (0.4)	0.61095
	SS	1.9 (1.4)	1.4 (1.1)	1.1 (0.7)	
Lactate levels, mmol/L	CS	4.9 (3.6)	1.2 (0.4)	1.2 (0.5)	0.84012
	SS	4.3 (2.4)	1.7 (0.8)	1.2 (0.5)	
SOFA	CS	11.0 (2.5)	7.5 (2.8)	5.2 (3.0)	0.36026
	SS	12.1 (2.0)	8.8 (3.0)	5.5 (3.3)	
Glasgow Coma Scale	CS	6 (4)	10 (3)	13 (3)	0.36746
	SS	5 (4)	9 (3)	11 (3)	
Platelets, 10^3^/mm^3^	CS	260 (85)	208 (80)	295 (138)	0.16299
	SS	205 (103)	176 (84)	235 (107)	
Hematocrit %	CS	39.2 (4.0)	34.9 (4.9)	35.2 (4.4)	0.00360
	SS	34.6 (5.0)	30.2 (5.2)	31.4 (4.4)	
Leukocytes total, 10^3^/mm^3^	CS	15.92 (5.36)	12.09 (4.20)	10.83 (2.93)	0.90718
	SS	16.19 (13.94)	14.55 (7.67)	13.66 (5.36)	
White blood cells, 10^9^/L	CS	15.29 (6.00)	12.2 (4.61)	11.29 (2.95)	0.98992
	SS	15.96 (14.22)	14.49 (7.95)	13.98 (5.59)	
Lymphocytes, 10^9^/L	CS	1.41 (0.70)	1.37 (0.65)	1.54 (0.67)	0.00007
	SS	0.70 (0.60)	0.80 (0.54)	0.90 (0.47)	
Neutrophils, 10^9^/L	CS	10.75 (6.27)	10.50 (5.95)	7.60 (1.38)	0.10346
	SS	14.49 (7.22)	11.32 (2.25)	12.14 (2.75)	

Clinical and laboratory characteristics of the patients divided by cardiogenic shock (CS) and septic shock (SS). Data are presented as mean (SD). *p* values were calculated with ANOVA and describe the significance of the difference between the variables in cardiogenic shock and septic shock over the three time points

**Table 2:** Clinical variables

Clinical variable	CS (*n* = 11)	SS (*n* = 21)	SC (*n* = 5)	*p* value SS vs CS	*p* value SS vs SC
Age, years	68.7 (13.0)	67.5 (19.2)	72.4 (15.8)	0.937	0.696
Body mass index, kg/m^2^	27.8 (6.9)	27.2 (5.5)	22.1 (6.9)	0.721	0.111
Sex, males	10 (90.9%)	14 (66.6%)	3 (60.0%)	0.210	1.000
Length of stay in ICU, days	7.6 (3.3)	9.2 (6.0)	6.0 (6.7)	0.920	0.046
Length of stay in hospital, days	24.2 (18.3)	28.0 (19.5)	28.0 (26.1)	0.676	0.696
APACHE II (T1)	22.73 (7.34)	24.57 (7.51)	14.6 (3.0)	0.450	0.012
SOFA (T1)	11.0 (2.5)	12.1 (2.0)	6.2 (2.2)	0.360	0.001
Mortality (28 days)	2 (18.2%)	5 (23.8%)	0 (0%)	1.000	0.545

Clinical characteristics of the patients divided by cardiogenic shock (CS), septic shock (SS), and septic controls (SC). Data are presented as mean (SD) or frequency (%). *p* values were calculated with Wilcoxon rank-sum test or alternatively with Fisher exact test for categorical variables

### Sequencing experiment

Total RNA libraries were sequenced in several batches, producing 28.61 M ± 6.49 M, 31.32 M ± 7.81 M, and 28.47 ± 4.37 raw read pairs on average for CS, SS, and SC groups, respectively. Ribosomal depletion was effective for all samples; the rRNA rate on mapped data was negligible in both groups (0.77 ± 1.25%, 0.80 ± 0.92%, and 1.38 ± 1.44% for CS, SS, and SC, respectively). The percentages of reads mapping to exons (85.20 ± 5.66% exonic rate) and DNase efficiency (3.02 ± 1.96% intergenic rate) were satisfactory in all samples. We obtained on average 12.19 ± 2.82, 14.12 ± 4.20, and 13.29 ± 1.62 million of uniquely and unambiguously mapped fragments for the CS, SS, and SC groups, respectively.

### Gene expression analysis over time

**Fig. 1:**
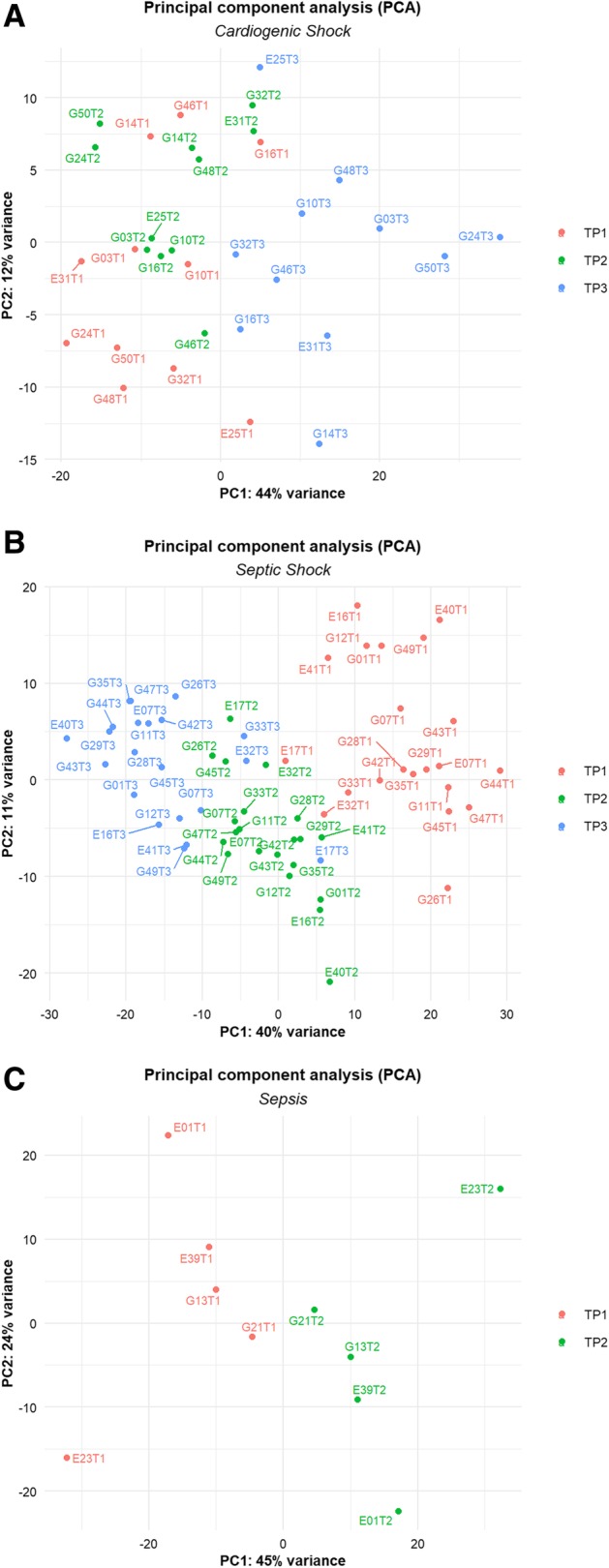
PCA plot of CS and SS patients. PCA plot of CS patients at three time points (**a**). PCA plot of SS patients at three time points (**b**). PCA plot of SC patients at T1 and T2 (**c**). PCs were adjusted in order to remove the patient effect

To explore whole gene expression modifications in CS, SS, and SC patients across the time points of the study, we first performed a principal component analysis (PCA) separately in each group of patients (Fig. 1a–c). PCA was performed on the 2000 most variable genes across samples. This analysis revealed that SS patients mostly clustered together according to time point on PC1, suggesting that the largest gene expression variation in the dataset was related to the time point (Fig. 1b). Also, in SC patients, we could appreciate a difference between the time points (Fig. 1c). In CS, on the contrary, patients clustered according to T3 only, but not to T1 and T2, suggesting minor gene expression changes between these two time points (Fig. 1a). Following this analysis, we independently identified genes that are differentially expressed (DEGs) in CS, SS, and SC patients over time. We found that SS patients modulated a higher number of genes compared to CS patients both in the comparison T2 vs T1 (CS = 12, SS = 3474) and T3 vs T1 (CS = 1073, SS = 6173). In the SC group, we detected 130 DEGs in the comparison T2 vs T1.

### Gene set enrichment analysis

**Fig. 2:**
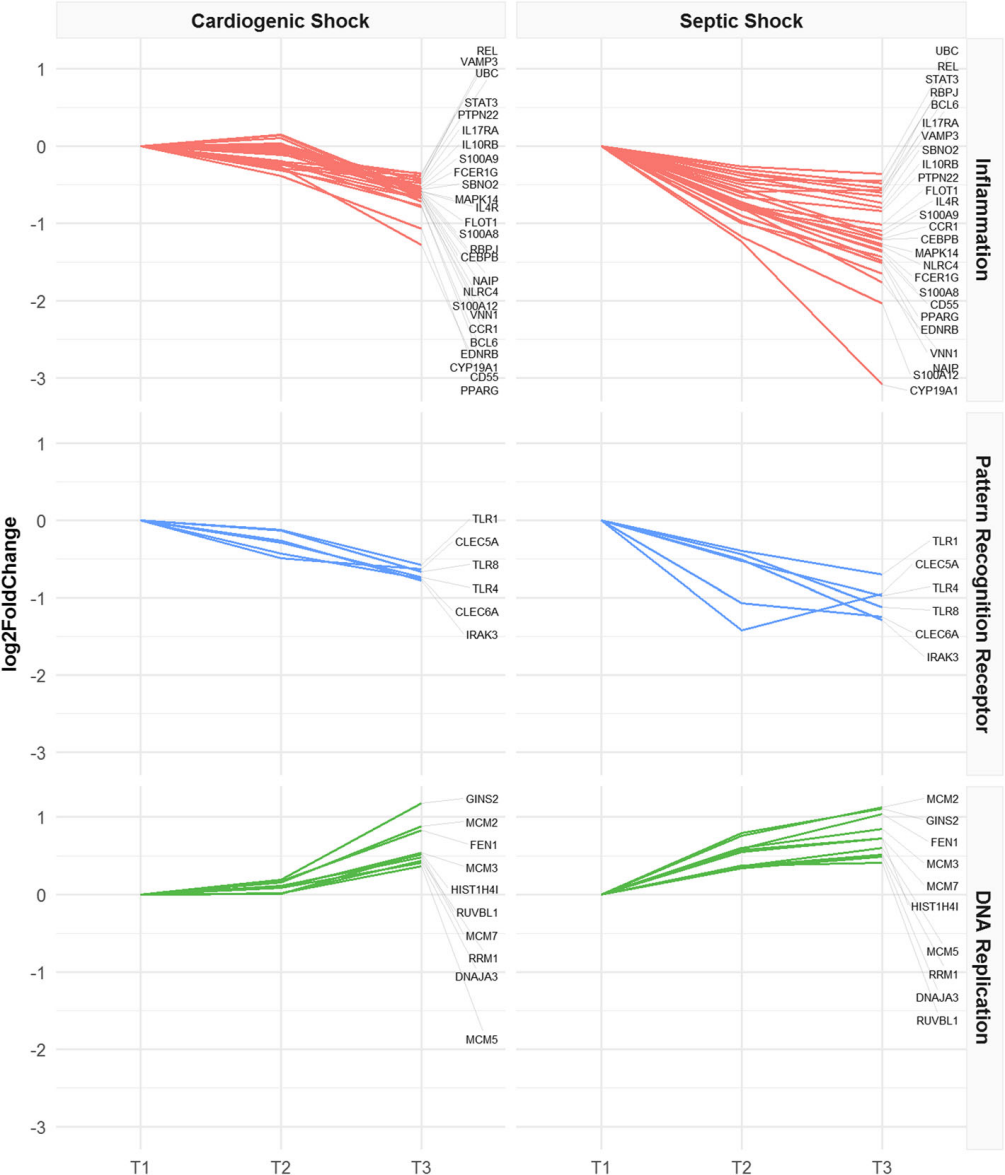
Inflammatory pathway, pattern recognition receptors, and DNA replication. Expression trends of significantly modulated genes in CS and SS, but not in SC patients. Data are normalized on T1; log_2_FoldChanges are plotted

Based on the exploratory analysis, we analyzed with GSEA the genes identified in each group, with the aim to pinpoint overrepresented classes of genes and Gene Ontology (GO) terms that describe the underlying biological processes. We used as input for GSEA the genes of the comparison between T1 and T2, separately in SS and SC. In CS, we analyzed the comparison T1 vs T3, because this is the time frame in which relevant gene expression differences were observed. We first selected GO terms significantly enriched in at least 1 type of shock, and we found a total of 315 downregulated and 78 upregulated biological processes (Additional file 4). GO terms of the inflammatory response and pattern recognition receptors (PRR) were downregulated over time, whereas GO terms related to DNA replication were upregulated in both CS and SS. We selected a list of inflammatory, PRR, and DNA replication genes showing significant modulation over time in CS and SS but not modulated in SC (Fig. 2, Table 3). In detail, we observed a negative expression trend for alarmins (S100A8, S100A9, S100A12), components of the inflammasome (NAIP, NLRC4), genes related to interleukin receptors (IL10RB, IL17RA, IL4R), transcription factors (CEBPB, PPARG, RBPJ, BCL6), Toll-like receptors (TLR1, TLR4, TLR8), and C-type lectin receptors (CLEC5A, CLEC6A). A positive expression trend was observed for genes essential for DNA replication (MCM2, MCM3, MCM5, MCM7). We also estimated the gene expression modifications in SS and CS comparing T1 to T3. In SS patients only, we found increasing expression of genes involved in defense response to bacteria and lymphocyte-mediated immunity, as well as decreasing expression of genes involved in platelet function and carbohydrate catabolic processes (Additional file 5, Additional file 8). Immunoglobulin genes encoding the heavy constant chains (IGHA1, IGHA2, IGHG1, IGHG2, IGHG3, IGHG4, IGHGP, IGHM) and the variable heavy and light chains (IGHV, IGKV, IGLV gene classes) were upregulated over 1 week of observation in both types of shock (Table 4).

**Table 3:** Gene expression changes in inflammatory pathway, pattern recognition receptors, and DNA replication

Gene name	Category	Molecular function	CS_log_2_FC_T1T3	SS_log_2_FC_T1T2
BCL6	Inflammation	Transcription factor	− 0.68	− 0.58
CCR1	Inflammation	Chemokine receptor	− 0.67	− 0.61
CD55	Inflammation	Regulation of complement cascade	− 0.77	− 1.00
CEBPB	Inflammation	Transcription factor	− 0.63	− 0.79
CYP19A1	Inflammation	Aromatase—estrogen synthesis	− 1.28	− 1.23
EDNRB	Inflammation	Endothelin receptor	− 0.79	− 0.90
FCER1G	Inflammation	Antibody receptor	− 0.55	− 0.80
FLOT1	Inflammation	Vesicle trafficking and cell morphology	− 0.60	− 0.77
IL10RB	Inflammation	Interleukin receptor	− 0.48	− 0.50
IL17RA	Inflammation	Interleukin receptor	− 0.47	− 0.35
IL4R	Inflammation	Interleukin receptor	− 0.58	− 0.82
MAPK14	Inflammation	MAPK p38	− 0.58	− 0.75
NAIP	Inflammation	Inflammosome	− 0.63	− 0.80
NLRC4	Inflammation	Inflammosome	− 0.63	− 0.73
PPARG	Inflammation	Transcription factor	− 1.07	− 0.78
PTPN22	Inflammation	Tyrosine phosphatase	− 0.43	− 0.65
RBPJ	Inflammation	Transcription factor	− 0.61	− 0.34
REL	Inflammation	NF-KB subunit	− 0.36	− 0.29
S100A12	Inflammation	Alarmin	− 0.71	− 1.17
S100A8	Inflammation	Alarmin	− 0.60	− 0.78
S100A9	Inflammation	Alarmin	− 0.53	− 0.54
SBNO2	Inflammation	Transcriptional regulation of NF-kappaB	− 0.56	− 0.38
STAT3	Inflammation	Transcription factor	− 0.42	− 0.43
UBC	Inflammation	Polyubiquitin precursor	− 0.38	− 0.26
VAMP3	Inflammation	Vesicular transport	− 0.35	− 0.46
VNN1	Inflammation	Leucocyte adhesion and migration	− 0.67	− 0.97
CLEC5A	Pattern recognition receptor	C-type lectin receptor	− 0.63	− 1.42
CLEC6A	Pattern recognition receptor	C-type lectin receptor	− 0.75	− 1.07
IRAK3	Pattern recognition receptor	PRR downstream signaling	− 0.78	− 0.51
TLR1	Pattern recognition receptor	Toll-like receptor	− 0.58	− 0.39
TLR4	Pattern recognition receptor	Toll-like receptor	− 0.74	− 0.53
TLR8	Pattern recognition receptor	Toll-like receptor	− 0.66	− 0.44
DNAJA3	DNA replication	Interactor of DNA polymerase	0.41	0.34
FEN1	DNA replication	DNA replication and repair	0.83	0.59
GINS2	DNA replication	DNA replication initiation	1.18	0.79
HIST1H4I	DNA replication	Replication-dependent histone	0.53	0.57
MCM2	DNA replication	MCM complex	0.88	0.76
MCM3	DNA replication	MCM complex	0.54	0.60
MCM5	DNA replication	MCM complex	0.36	0.36
MCM7	DNA replication	MCM complex	0.48	0.55
RRM1	DNA replication	Biosynthesis of deoxyribonucleotides	0.43	0.36
RUVBL1	DNA replication	DNA helicase	0.51	0.37

Genes with significant modulation over time in both CS and SS, but not in SC. Description of functions and log_2_FoldChange expression are shown

**Table 4:** Gene expression changes of immunoglobulins

Gene ID	Gene name	Chain	Region	Antibody class	CS_log_2_FC_T1T3	SS_log_2_FC_T1T3
ENSG00000211895	IGHA1	Heavy	Constant	IgA	1.25	1.48
ENSG00000211890	IGHA2	Heavy	Constant	IgA	1.27	1.52
ENSG00000211896	IGHG1	Heavy	Constant	IgG	1.05	1.15
ENSG00000211893	IGHG2	Heavy	Constant	IgG	1.28	2.15
ENSG00000211897	IGHG3	Heavy	Constant	IgG	1.14	1.28
ENSG00000211892	IGHG4	Heavy	Constant	IgG	0.95	1.52
ENSG00000253755	IGHGP	Heavy	Constant	IgG	0.99	1.30
ENSG00000211899	IGHM	Heavy	Constant	IgM	0.98	0.84
ENSG00000211934	IGHV1-2	Heavy	Variable	–	0.92	1.25
ENSG00000211935	IGHV1-3	Heavy	Variable	–	0.91	1.13
ENSG00000211962	IGHV1-46	Heavy	Variable	–	0.72	1.17
ENSG00000211942	IGHV3-13	Heavy	Variable	–	0.89	1.36
ENSG00000211943	IGHV3-15	Heavy	Variable	–	1.37	1.62
ENSG00000211947	IGHV3-21	Heavy	Variable	–	1.11	1.25
ENSG00000211949	IGHV3-23	Heavy	Variable	–	1.35	1.30
ENSG00000270550	IGHV3-30	Heavy	Variable	–	1.10	1.17
ENSG00000211964	IGHV3-48	Heavy	Variable	–	1.09	1.15
ENSG00000211965	IGHV3-49	Heavy	Variable	–	0.87	1.22
ENSG00000282639	IGHV3-64D	Heavy	Variable	–	1.11	1.31
ENSG00000211938	IGHV3-7	Heavy	Variable	–	1.43	1.65
ENSG00000225698	IGHV3-72	Heavy	Variable	–	1.73	1.46
ENSG00000211976	IGHV3-73	Heavy	Variable	–	1.11	1.73
ENSG00000224650	IGHV3-74	Heavy	Variable	–	1.24	1.56
ENSG00000211959	IGHV4-39	Heavy	Variable	–	0.87	1.08
ENSG00000276775	IGHV4-4	Heavy	Variable	–	0.93	1.26
ENSG00000224373	IGHV4-59	Heavy	Variable	–	1.11	1.05
ENSG00000211966	IGHV5-51	Heavy	Variable	–	0.85	1.00
ENSG00000211933	IGHV6-1	Heavy	Variable	–	1.25	1.16
ENSG00000211592	IGKC	Light	Constant	–	1.30	1.48
ENSG00000211597	IGKJ1	Light	Joining	–	0.89	1.21
ENSG00000211594	IGKJ4	Light	Joining	–	0.89	1.19
ENSG00000243290	IGKV1-12	Light	Variable	–	1.28	0.94
ENSG00000240864	IGKV1-16	Light	Variable	–	1.11	1.01
ENSG00000240382	IGKV1-17	Light	Variable	–	1.26	1.43
ENSG00000244575	IGKV1-27	Light	Variable	–	1.21	1.05
ENSG00000243466	IGKV1-5	Light	Variable	–	1.10	1.39
ENSG00000239855	IGKV1-6	Light	Variable	–	1.07	1.65
ENSG00000241755	IGKV1-9	Light	Variable	–	1.08	1.17
ENSG00000241294	IGKV2-24	Light	Variable	–	1.13	1.71
ENSG00000243238	IGKV2-30	Light	Variable	–	1.24	1.50
ENSG00000243264	IGKV2D-29	Light	Variable	–	1.10	1.32
ENSG00000241351	IGKV3-11	Light	Variable	–	1.43	1.14
ENSG00000244437	IGKV3-15	Light	Variable	–	1.08	1.23
ENSG00000239951	IGKV3-20	Light	Variable	–	1.20	1.46
ENSG00000211625	IGKV3D-20	Light	Variable	–	1.02	1.31
ENSG00000211598	IGKV4-1	Light	Variable	–	1.27	1.72
ENSG00000211677	IGLC2	Light	Constant	–	1.26	1.52
ENSG00000211679	IGLC3	Light	Constant	–	1.32	1.45
ENSG00000211642	IGLV10-54	Light	Variable	–	1.09	1.17
ENSG00000211653	IGLV1-40	Light	Variable	–	1.05	1.21
ENSG00000211651	IGLV1-44	Light	Variable	–	0.97	1.32
ENSG00000211648	IGLV1-47	Light	Variable	–	1.03	1.63
ENSG00000211644	IGLV1-51	Light	Variable	–	1.16	1.24
ENSG00000211668	IGLV2-11	Light	Variable	–	1.08	1.57
ENSG00000211666	IGLV2-14	Light	Variable	–	1.21	1.62
ENSG00000211660	IGLV2-23	Light	Variable	–	1.21	1.70
ENSG00000278196	IGLV2-8	Light	Variable	–	1.44	1.57
ENSG00000211673	IGLV3-1	Light	Variable	–	0.79	1.15
ENSG00000211663	IGLV3-19	Light	Variable	–	0.82	1.21
ENSG00000211662	IGLV3-21	Light	Variable	–	0.89	1.08
ENSG00000211637	IGLV4-69	Light	Variable	–	0.94	1.19
ENSG00000211650	IGLV5-45	Light	Variable	–	0.83	0.96
ENSG00000211640	IGLV6-57	Light	Variable	–	1.08	1.33
ENSG00000211652	IGLV7-43	Light	Variable	–	1.13	1.65
ENSG00000211649	IGLV7-46	Light	Variable	–	1.06	1.76
ENSG00000211638	IGLV8-61	Light	Variable	–	1.13	1.62

Genes with significantly different expression in both CS and SS over 1 week. Description of functions and log_2_FoldChange expression are shown

### Analysis of transcription factor target genes

**Fig. 3:**
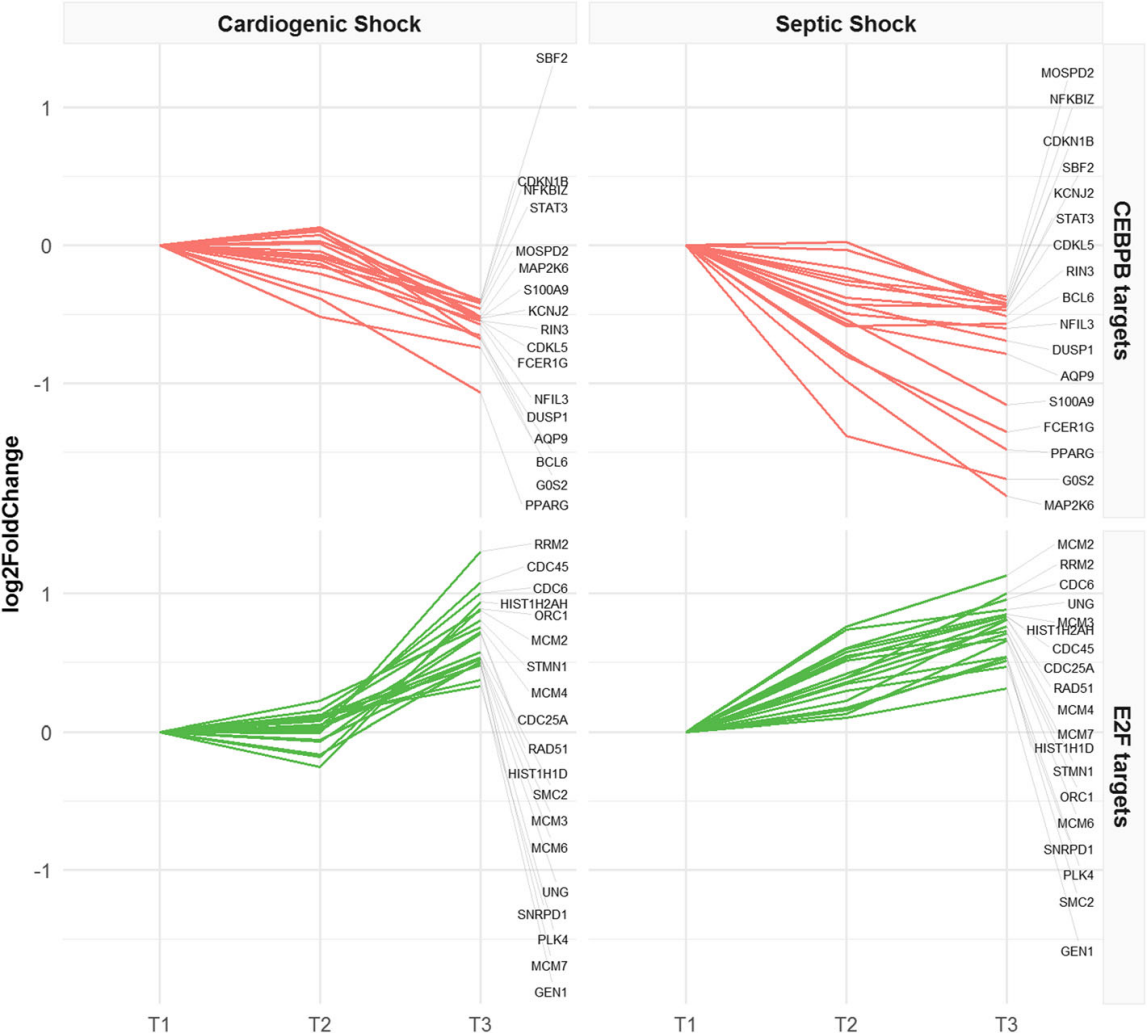
Gene expression trends of enriched transcription factor targets in CS and SS. CEBPB and E2F target genes are shown. Data are normalized on T1; log_2_FoldChanges are plotted

We searched for overrepresented transcription factor targets in the lists of genes ranked according to log_2_FC in the CS and SS groups separately. GSEA results are as shown in Additional file 6. Then, we focused on the common enriched transcription factors: we observed that genes with a negative expression trend were enriched in target sequences of CCAAT-enhancer-binding protein beta (CEBPB), whereas positively regulated genes were enriched in target sequences of the members of the E2F family of transcription factors (Table 5, Fig. 3).

**Table 5:** GSEA of transcription factor targets (TFT)

Gene set (TFT)	CS_NES	SS_NES	CS_FDR.q.val	SS_FDR.q.val	DEGs_CS	DEGs_SS	Trend
CEBPB_02	− 1.80	− 1.87	0.08739	0.02342	23	56	Negative
E2F_Q3_01	1.99	1.64	0.00111	0.02955	20	54	Positive
E2F_Q4_01	1.89	1.87	0.00144	0.01000	20	57	Positive
E2F_Q6	1.95	1.55	0.00117	0.06391	18	58	Positive
E2F_Q6_01	1.93	1.65	0.00128	0.03002	20	54	Positive
E2F1_Q4_01	1.92	1.63	0.00123	0.03049	20	52	Positive

For each gene set of TFT is reported: normalized enriched score, false discovery rate, the number of target genes differentially expressed in CS and SS, and the gene expression trend

## Discussion

This study describes the transcriptome profile of circulating cells in CS and SS patients over the first week after ICU admission, using septic patients without shock as controls. The study design is based on three time points (T1, T2, T3) over 1 week of observation in shock patients and two time points (T1, T2) in septic controls. To our knowledge, an analysis with this time frame from shock onset is new in the field of SS research, and this is the first transcriptomic study in CS.

At study enrollment, illness severity, evaluated as SOFA score and lactate level, was comparable between CS and SS patients and decreased during 1 week of ICU stay. The dosage of norepinephrine needed to maintain blood pressure was also similar between the two groups, demonstrating a comparable degree of vasoplegia. However, significant differences were found in the levels of C-reactive protein and lymphocyte count that could be explained by the pathophysiology of the two types of shock.

**Fig. 4:**
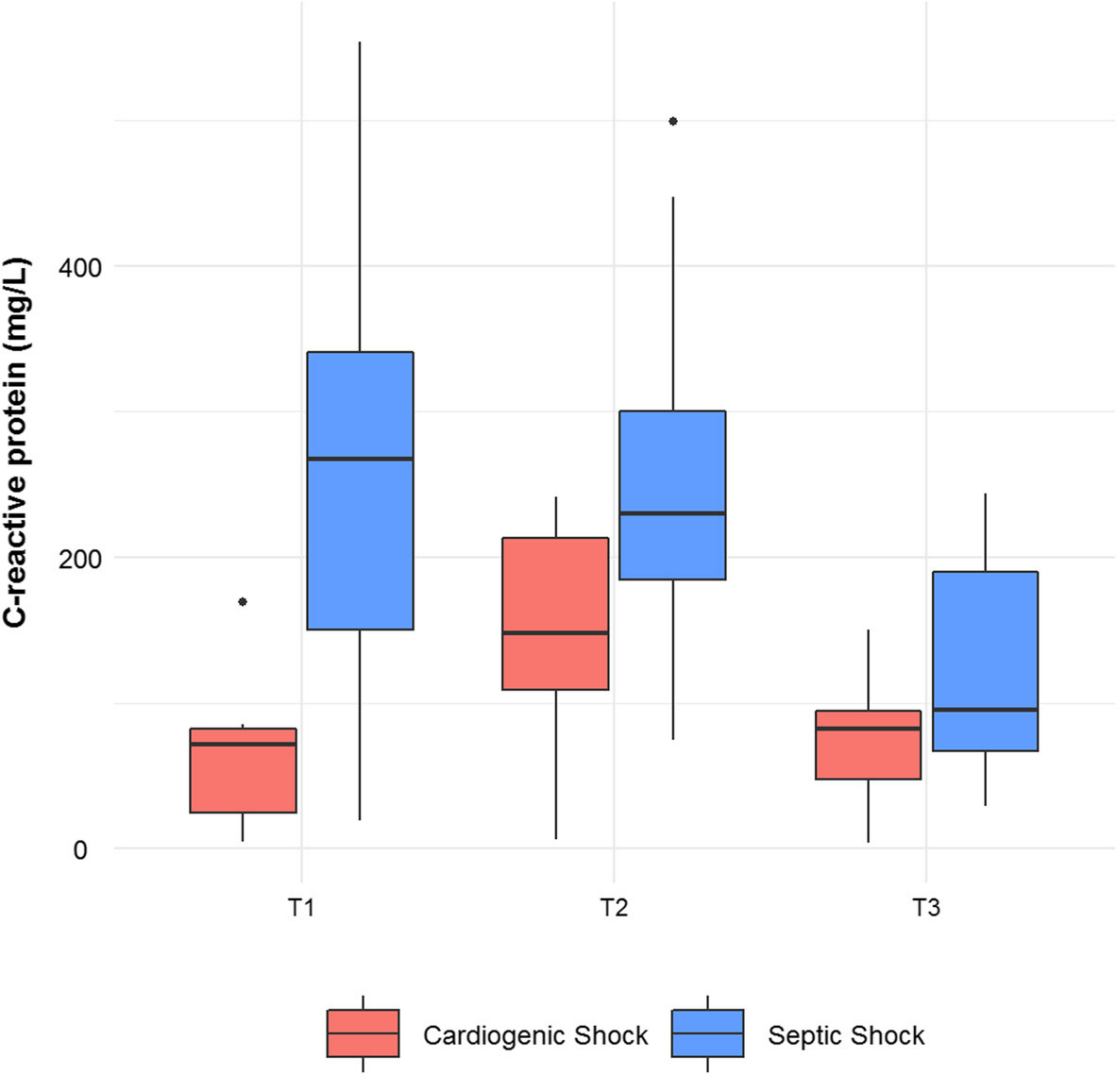
Boxplots of C-reactive protein serum measurements. C-reactive protein measurements (mg/L) in cardiogenic and septic shock patients measured at three time points. The lower and upper hinges correspond to the 25th and 75th percentiles, respectively

Within-group transcriptomic analysis over time showed differences in the number of genes modulated in SS and CS during the period of observation, with a larger number of differentially expressed genes identified in SS compared to CS. The timing of gene expression modulation was different as well, compared to the time of shock diagnosis (T1). SS patients showed significant modifications both in the early phase that corresponds to T2 and after 7 days of ICU stay (T3), whereas in CS patient, relevant and significant transcriptomic changes occurred only at T3. Differential expression in these time frames identified genes and pathways common to both CS and SS groups, but not found in SC. Among the genes identified in both shock groups, GSEA highlighted a negative expression trend for genes involved in inflammatory processes including alarmins, inflammasome, and interleukin receptors, implying that the acute phase of shock in these patients was characterized by an inflammatory peak that decreased after supportive treatment. From our results, it appears that at the transcriptional level inflammatory processes are more rapidly downregulated in SS (Fig. 2) and more slowly in CS patients. A different timing of the inflammatory processes in CS and SS was also suggested by the trend of CRP serum levels in the three time points (Fig. 4). A similar time course of the expression of C-reactive protein has been previously described by Parenica et al. [20].

In both CS and SS patients, SOFA scores showed a decreasing trend in time, suggesting a link between acute inflammation and organ dysfunction as it has been previously found in acute illness [21]. Pattern recognition receptors (PRRs), including Toll-like receptors (TLRs) and C-type lectin receptors, were downregulated after 1 week in both shock types. PRRs are essential for the early detection of pathogens and the initiation of an adequate innate immune response [22, 23], and they play a well-known role in the development and pathogenesis of sepsis [24]. PRRs can also be activated by damage-associated molecular patterns (DAMPs) [25–27], which act as triggers of inflammation, cell injury, vascular leakage, and multiple organ dysfunction in acute illness [24, 28, 29]. This may be a possible explanation for the inflammatory modulation observed in CS patients, as the involvement of pattern recognition receptor signaling and inflammatory mediators has been documented in the pathogenesis of heart failure [30–32] and lung injury [33]. Transcription factors with inflammation-related associations also displayed a decreasing expression trend, including peroxisome proliferator-activated receptor gamma (PPARG), a regulator of inflammation and the lipid pathway, and CCAAT-enhancer-binding protein beta (CEBPB), which is necessary for normal macrophage inflammatory response [34]. Decreases in these inflammation-related transcription factors correlated significantly with the decreased expression of a set of genes related to inflammation and immunity.

During the critical illness period from T1 to T3, we observed a positive expression trend of DNA replication genes (Fig. 2) in both SS and CS groups, suggesting that during shock, at least, a subset of circulating cells undergoes a process of self-renewal. In this time frame, we also observed the upregulation of immunoglobulins, implying an activation of the adaptive immune system that is co-existent with innate immune system downregulation. Consistent with our observations, low serum levels of circulating immunoglobulins have been described at septic shock onset [35–37], and Venet et al. observed their increase to reference levels after 5–7 days [38]. Interestingly, the increasing trend of immunoglobulin gene transcription in SS was also observed in CS patients, suggesting that Igs may also have a role in the absence of infection. This is in agreement with the findings of Andaluz-Ojeda et al. who investigated the role of Ig in non-septic critically ill patients [39]. The role of Igs in the absence of infection may be related both to the detection of DAMPs [40] and to the immunomodulatory activity of the adaptive immune response [41]. The analysis of transcription factor targets in the CS and SS groups revealed a significant enrichment of genes regulated by the E2F transcription factor family, which are involved in the control of cell cycle progression and cell proliferation [42, 43].

In this study, we could also focus on genes that modify their expression uniquely in SS. In these patients, as expected, specific transcriptomic modifications were observed in genes involved in the response to infection, lymphocyte-mediated immunity, carbohydrate metabolism, and platelet function. These processes are implicated in the pathophysiology of sepsis and have already been described in previous works [44–46]. In CS patients, our study was unable to detect any specifically modulated pathway in the blood cells; transcriptional modifications observed in this group were associated with biological functions common to SS.

The present work has two limitations. The first is the small sample size, which could hamper the detection of small, but still relevant gene expression changes. Moreover, studies of large cohorts [47] and the poor success of the past clinical trials [48] showed that sepsis is a heterogeneous condition, with high between-patient variability, and looking at a small cohort of patients make difficult the identification of different phenotype subgroups. The same concept is extendible to cardiogenic shock patients, as they also could show phenotypes or different heart failure and circulatory shock mechanisms [32]. To partially overcome between-patient variability, we used a paired analysis to assess the gene expression changes, which takes advantage of the within-patient correlation between the time points, strengthens the analysis, and improves the statistical power.

A second limitation is that, since we used a study design based on three time points, we have excluded the most severe patients who died before the second or third time point. Thus, the results of our study describe only patients surviving at least 1 week, whereas patients who died early could have different expression signals. The exclusion of the most severe patients can also explain why mortality in our shock patients was low in comparison with broader modern clinical trials or large surveys. In addition, in our cardiogenic shock patients, the amount of CS not related to an acute coronary syndrome was 7/11 (Additional file 1), a condition that is associated with a mortality rate < 25% as reported by Harjola et al. [49].

In short, our study pinpoints a common modulation in SS and CS patients of genes of inflammation, PRRs, DNA replication, and immunoglobulins, irrespective of the etiology. These pathways have been previously investigated in SS, and their perturbation can be interpreted as the response of the immune system to a widespread infection. Their modulation in CS patients suggests that their role may be independent of infection and sepsis and should rather be seen in the context of dysfunctions associated with circulatory shock. The overlap in molecular patterns observed in this study suggests shared mechanistic pathways between CS and SS. This could help to identify common targets for more personalized therapies that can be used in different critical illness conditions [50], as previously demonstrated and is ongoing in cancer research [51].

## Conclusions

Our preliminary results support a central role for acute inflammatory processes in the pathophysiology of shock, with the hypothesis that pattern recognition receptors, alarmins, and immunoglobulins may serve as mediators. This study has the limit of a small sample size. However, it encourages new –omics studies in larger cohorts of circulatory shock patients to investigate the relationships of PRRs, inflammation, and immunoglobulins with outcomes.

## Supplementary information

**Additional file 1: Table S1.** Description of CS, SS and SC patients included in the study. For each patient is reported the type of shock, gender and age, mortality at 28 days. The cause for CS is specified for CS patients, whereas for SS and SC patients the source of infection is indicated.

**Additional file 2: Figure S1.** Flow Chart of the process of patient recruitment. Description of the process of selection of the patients included in the study.

**Additional file 3: Figure S2.** SOFA score trends in CS and SS patients according to mortality. Boxplots of SOFA scores evaluated at three timepoints in CS and SS patients according to the mortality at 28 days.

**Additional file 4.** Results of GSEA of Gene Ontologies in CS and SS. Upregulated (Excel Sheet “GSEA_UP”) and downregulated (Excel Sheet “GSEA_DOWN”) gene sets resulting from the GSEA analysis in CS, SS and SC. For each significantly enriched GO is reported the number of DEGs, the Normalized Enriched Score (NES), the False Discovery Rate (FDR.q.val).

**Additional file 5: Figure S3.** Gene expression trends of biological processes enriched only in SS patients. Gene expression trends of biological processes related to defense response to bacterium, lymphocyte mediate immunity, platelet activation and degranulation, carbohydrate catabolic process. Data are normalized on T1, log_2_FoldChanges are plotted.

**Additional file 6.** Results of the GSEA of Transcription Factor Targets (TFT). Results table of the GSEA analysis of genes ranked for log_2_FC over one week in CS and SS. For each gene set of TFT is reported: number of DEGs and number of genes in the gene set (SIZE), Normalized Enriched Score, False Discovery Rate and type of shock.

**Additional file 7. **Clinical variables of SS and SC patients. Clinical characteristics of the patients divided by Septic Shock and Septic Controls. Data are presented as mean (SD). *P*-values were calculated with ANOVA and describe the significance of the difference between the variables in Septic Shock and Septic Controls over 2 timepoints.

**Additional file 8.** Gene expression trends of biological processes enriched only in SS patients. Extensive information of genes shown in Figure S3 is reported in this table. Data are normalized on T1, log_2_FoldChanges are shown.

## Data Availability

The datasets generated and analyzed during the current study are available through the Gene Expression Omnibus Database (accession number GSE131411).

## Abbreviations

ICU: Intensive care unit
SS: Septic shock
CS: Cardiogenic shock
SC: Septic controls (not developing shock)
SOFA: Sequential Organ Function Assessment
DEG: Differentially expressed gene
FDR: False discovery rate
GSEA: Gene set enrichment analysis
GO: Gene Ontology
BMI: Body mass index
PCA: Principal component analysis
T1: Time point 1
T2: Time point 2
T3: Time point 3
Ig: Immunoglobulin
PRR: Pattern recognition receptor
DAMP: Damage-associated molecular pattern
TLR: Toll-like receptor
