# Supplementary figures and images for: A longitudinal study highlights shared aspects of the transcriptomic response to cardiogenic and septic shock
Source: Crit Care. 2019 Dec 19;23:414. doi: 10.1186/s13054-019-2670-8 (PMC6921511; doi:10.1186/s13054-019-2670-8)

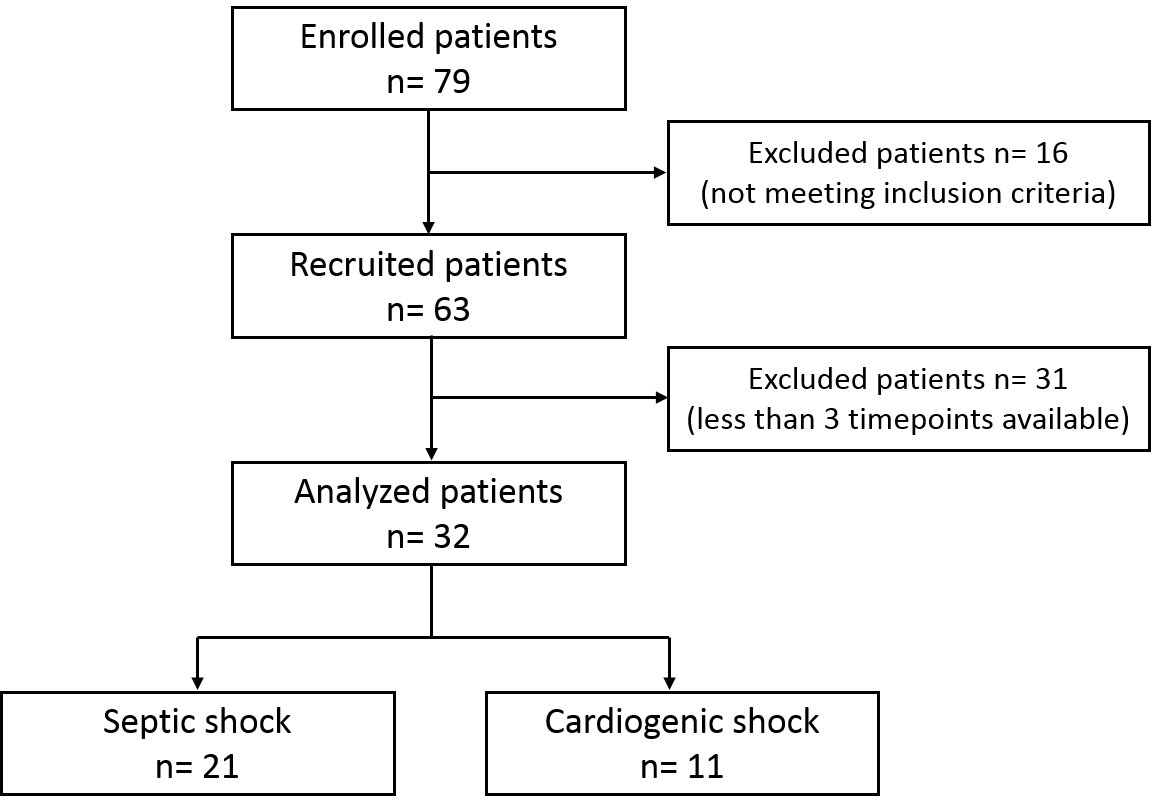

Supplement: Supplementary file 2 — Additional file 2: Figure S1. Flow Chart of the process of patient recruitment. Description of the process of selection of the patients included in the study. [file 13054_2019_2670_MOESM2_ESM.png]

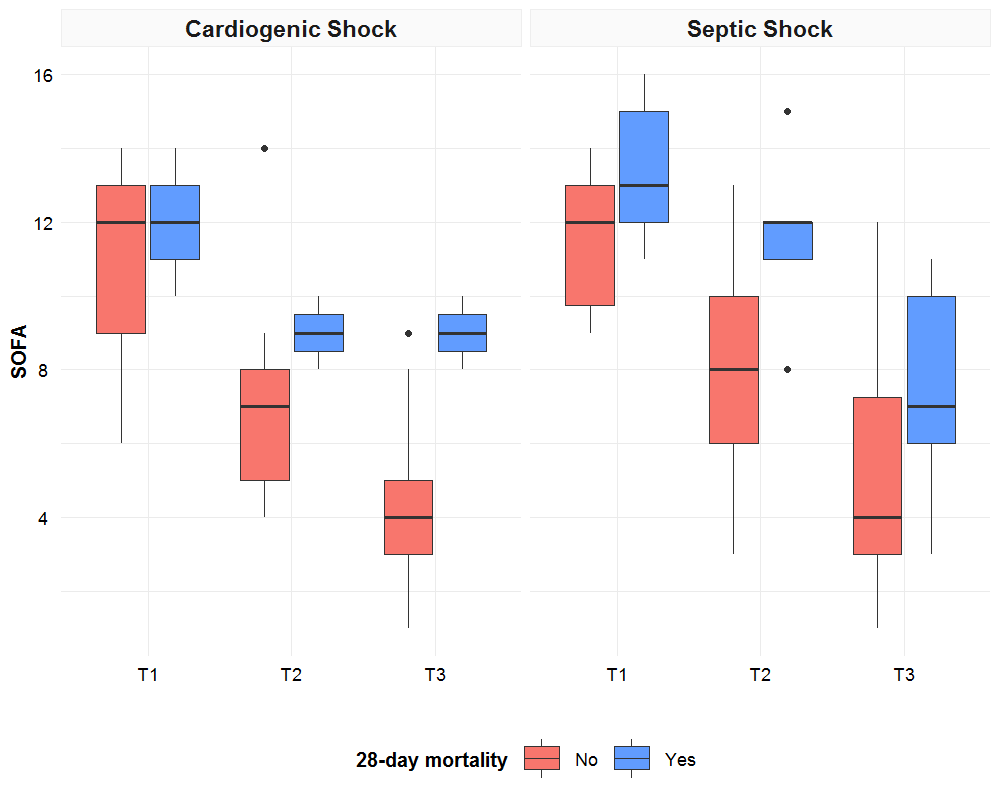

Supplement: Supplementary file 3 — Additional file 3: Figure S2. SOFA score trends in CS and SS patients according to mortality. Boxplots of SOFA scores evaluated at three timepoints in CS and SS patients according to the mortality at 28 days. [file 13054_2019_2670_MOESM3_ESM.png]

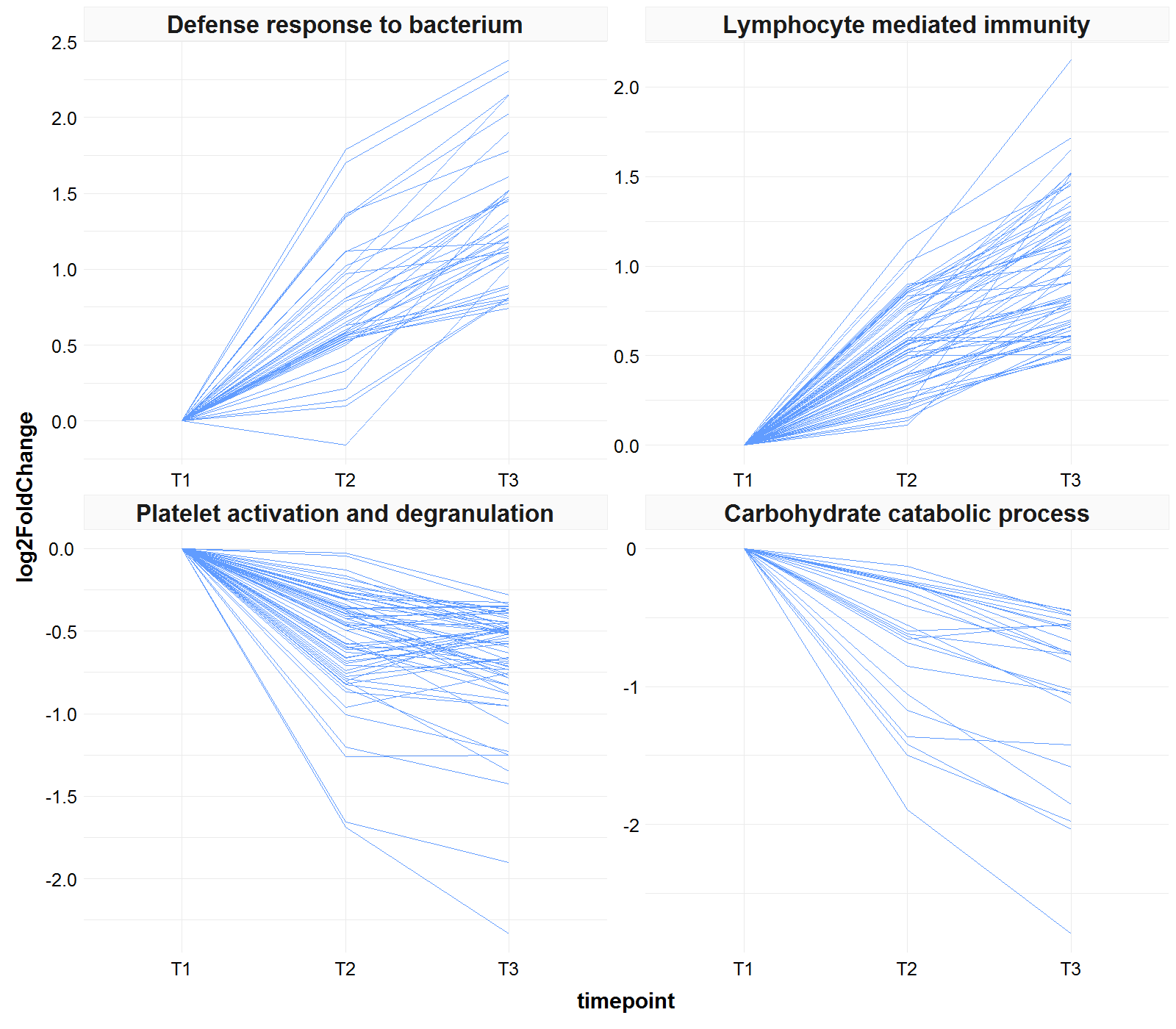

Supplement: Supplementary file 5 — Additional file 5: Figure S3. Gene expression trends of biological processes enriched only in SS patients. Gene expression trends of biological processes related to defense response to bacterium, lymphocyte mediate immunity, platelet activation and degranulation, carbohydrate catabolic process. Data are normalized on T1, log2FoldChanges are plotted. [file 13054_2019_2670_MOESM5_ESM.png]
